# Factors affecting the follow-up time after a positive result in the fecal occult blood test

**DOI:** 10.1371/journal.pone.0258130

**Published:** 2021-10-05

**Authors:** Yin-Wen Cheng, Ying-Chun Li

**Affiliations:** 1 Department of Business Management, College of Management, National Sun Yat-Sen University, Kaohsiung, Taiwan, R.O.C; 2 Institute of Health Care Management, National Sun Yat-Sen University, Kaohsiung, Taiwan, R.O.C; Chang Gung Memorial Hospital and Chang Gung University, Taoyuan, TAIWAN

## Abstract

In 2010, Taiwan included the fecal occult blood test (FOBT) under preventive health insurance services. For patients whose test positive, receiving follow-ups is paramount. This study investigated factors affecting the follow-up time of these patients. This retrospective study used data from the colorectal cancer screening archives. The study period was from 2010 to 2013, and the subjects were 50–75-year-old persons who tested positive for FOBT. The t test, one-way ANOVA, and multiple regression were performed to address the differences in the mean tracking period between variables such as the population’s demographic characteristics. The mean follow-up time for the 98,482 participants whose screening results were positive exhibited significant differences (p < 0.001) according to medical unit region and classification, age, screening location, family history, examination method, and diagnosis. The model predicting the mean follow-up time predicted a period of 10.079 days longer for those whose hospital was on an offshore island than that of those whose hospital was in the eastern regions. The follow-up time was 1.257 days shorter for people who were inpatients than those who were outpatients and was 8.902 days longer for people who underwent double contrast barium enema plus flexible sigmoidoscopy than those who underwent other examination methods. Patients with a family history of colorectal cancer and those whose examination results indicated cancer had a follow-up time of 2.562 and 2.476 days shorter than those who did not know their family history and those with other results, respectively. Factors affecting the follow-up time of people whose FOBT results were positive consisted of the location and classification of the follow-up institution, age, screening location, family history, examination method, and diagnosis. This provides valuable references for improving the cancer screening program.

## Introduction

Since 1982, the main cause of death in Taiwan has changed from acute diseases to cancer. Among cancers, colorectal cancer (CRC) exhibited the most substantial change. According to the Death Statistics from the Ministry of Health and Welfare and the Cancer Registration Annual Report published in 2018, among the ten most prevalent cancers in Taiwan, the number of cases of CRC was 16,525, and the age-standardized incidence was 41.84/100,000. The number of deaths was 5,823, and the age-standardized deaths were 13.78/100,000. CRC ranked third in the list of causes of death because of cancer [1,2]. The aforementioned data indicate that CRC is one of the major causes of death and of cancer condition.

According to statistics from the Global Cancer Observatory, in 2020, the number of cases of CRC worldwide was 1,931,590, and the number of mortalities was 935,173. When the data were analyzed based on continent and age, the number of CRC cases aged between 50 and 74 years of age was highest in Asia (635,428 cases) followed by Europe (295,645). The number of CRC mortalities between 50 and 74 years of age was also highest in Asia (269,966 cases) followed by Europe (110,338) [3]. Scholars have stated that lifestyle, ethnicity, and genetic factors affect the incidence of CRC, and these factors lead to large differences in countries worldwide [4–6]. The aforementioned international statistics and research results reveal that CRC incidence and deaths are closely related to region, ethnicity, and age.

Due to the burden and threat that CRC poses on health, the Cancer Control Act in Taiwan has, since 2010, included the fecal occult blood test (FOBT) under preventive health care services. The method employed is the fecal immunochemical test (FIT), and the recipients are people aged 50–75 years [7,8]. If the test result is positive, then follow-up examinations should be arranged. In Israel, the Netherlands, Denmark, Canada, and the United States, the FOBT or colonoscopy are offered to high-risk groups [9–15]. Clearly, each country values the prevention and treatment of CRC. Currently, Taiwan and other countries consider colonoscopy as an ideal tool for diagnosis.

A potential problem is that many people testing positive in the FOBT cannot be effectively traced. Relevant studies have reported that 40%–60% of people who underwent the FOBT and tested positive could not be traced [16,17]. Another large-scale community study revealed that people whose FOBT result was positive and who underwent colonoscopy more than 6 months after the test have an increased risk of CRC-related or terminal diseases [18]. Testing positive in the FOBT involves possible changes of the disease; thus, identification of factors affecting the follow-up time are critical [19,20]. Therefore, this study explored factors affecting the follow-up of cases with positive FOBT results to understand why follow-up delays occur.

Recent studies on screening for and following up CRC have mainly adopted insurance database analysis, case review, and questionnaire survey methods [9,19,21–24]. Few health authorities have purposefully established a database for people participating in CRC screening containing relevant data from across the whole country. Therefore, this study aimed to discuss the effects of demographic features, cancer screening factors, examination methods, and diagnosis results of cases testing positive for FOBT on mean follow-up time. The results may serve as references for future studies and formulation of cancer prevention policies.

## Materials and methods

### Research design

A retrospective study design was adopted, and the study period was from 2010 to 2013. The participants were people first receiving FOBT for preventive health care.

### Materials and participants

**Fig 1:**
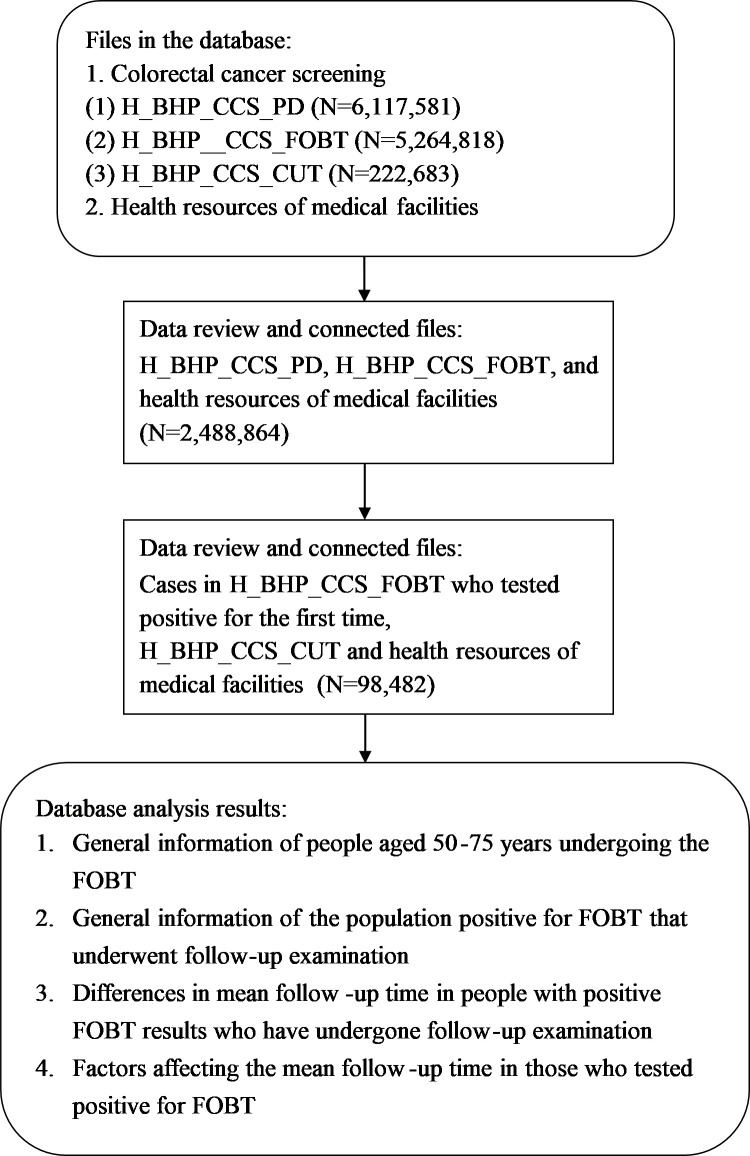
Data review and analysis procedure.

The database consisted of cancer screening—CRC screening data (H_BHP_CCS) and information on health resources of medical facilities (H_OST_RESMF) maintained by the Health and Welfare Data Science Center of the Ministry of Health and Welfare (HWDC) [25,26]. The participants were people aged 50–75 years who underwent the FOBT for the first time and were found to be positive for FOBT. Colorectal cancer screening data includes the following: 1. colorectal cancer screening-general information data (H_BHP_CCS_PD) consisting of data on the population receiving preventive health care that met the criteria for undergoing the FOBT. There were 6,117,581 datasets in the database. 2. FOBT data (H_BHP_CCS_FOBT) consisting of data on the people who actually underwent FOBT, and the screening method used for all subjects was FIT. There were 5,264,818 datasets in the database. 3. colonoscopy report (H_BHP_CCS_CUT) data consisting of data on people with positive FOBT reports who underwent colonoscopy. The number of datasets in the database was 222,683 [25]. Data were reviewed and linked according to the study objective, and their contents are shown in Fig 1. “Positive cases with completed follow-up” were defined as people who tested positive for FOBT and, during the follow-up period, underwent colonoscopy, double contrast barium enema plus flexible sigmoidoscopy, or other recheck methods. “FOBT” refers to the FIT provided under preventive health care services.

### Operational definitions of variables

FOBT results were defined as “negative or positive.” Independent variables consisted of the following. Sex was defined as “male or female.” The locations of medical institutions for screening and the follow-up examination were defined as “offshore cities and counties, northern region, central region, southern region, or eastern region” [27]. The age groups that were screened and tested positive were classified as “50, 51–55, 56–60, 61–65, 66–70, and 71–75 years old.” Screening locations were defined as “community or workplace screening station, outpatient, inpatient, or other.” Categories of medical units where screening and follow-up examination were performed were classified as “outpatient and inpatient”, of which “outpatient” referred to clinics and outpatient departments of hospitals. The definition of family history of CRC was “no, yes, or unknown.” Examination methods were defined as “colonoscopy, double contrast barium enema plus flexible sigmoidoscopy, or others.” Test results were defined as “normal, hemorrhoids, ulcerative colitis, polyp, CRC, or others.” The dependent variable was the mean follow-up time (calculated in days), specifically, the period between the receipt of the positive FOBT result report and the completion of the colonoscopy, double contrast barium enema plus flexible sigmoidoscopy, or other examination method.

### Data analysis

#### Data review

First, “colorectal cancer screening-general information data” in the colorectal cancer screening data (N = 6,117,581) was reviewed to obtain the total number, gender, and year and month of birth of subjects who could undergo the FOBT. The “colorectal cancer screening-FOBT” (N = 5,264,818) data was inspected in terms of gender, screening region, screening location, medical institution code, and family history of colorectal cancer. Missing values and wrongly coded values were checked or deleted. Furthermore, subjects with outpatient visit date and examination date between 2010 and 2013 and those who underwent the first screening were selected as study subjects, and different databases were linked according to the study objective. These databases include “colorectal cancer screening-general information data”, “colorectal cancer screening-FOBT,” and health resources of medical facilities. After data processing, the total number of cases was 2,488,864. After analysis, general information regarding the 50–75 years old cohort that underwent the first FOBT was obtained. Following that, the data of 191,671 positive cases on screening were inspected and cross-validated with the “colorectal cancer screening-colonoscopy report” (N = 222,683) and health resources of medical facilities. The data was examined for screening criteria and study period (19%), removal of missing and unknown labels (21%), and unreasonable test report date and follow-up examination date (8.6%). These data were excluded from the study. Finally, 98,482 people with positive FOBT results who underwent follow-up examination were obtained. This data was then analyzed and processed to identify differences in general information and the mean follow-up time of people with positive FOBT results across different study variables and to analyze factors that affect the mean follow-up time in these people (Fig 1).

#### Descriptive statistics

FOBT result, sex, screening and medical institution region where the follow-up was performed, age at positive result and screening, screening location, medical unit category where screening and follow-up examination was performed, family history of CRC, examination method, diagnosis, and follow-up time are presented as percentage distributions and as means.

### Inferential statistics

1. Independent sample *t* test and one-way analysis of variance were used to analyze the sex, hospital region where the follow-up was performed, age, screening location, medical unit category where the follow-up was performed, family history of CRC, examination method, and diagnosis results of the participants to determine whether differences existed in mean follow-up time.
2. Multiple regression analysis was performed to assess the influence of sex, region of the medical institution where the follow-up was performed, age at positive result, screening location, medical unit category where the follow-up was performed, family history of CRC, examination method, and diagnosis results factors affecting the mean follow-up time.

The statistical analysis software used was IBM SPSS Statistics 21.

### Ethics statement

This study was reviewed and approved by the Institutional Review Board of Antai Medical Care Cooperation Antai Tian-Sheng Memorial Hospital in 2019, and the review number was 19-057-C. The study application for the use of data from the colorectal cancer screening and health resources of medical facilities databases was also approved, and the application number was H108141. The database content in this study was protected by the Personal Data Protection Act and Human Subjects Research Act and have been de-identified and de-linked, coded, and encrypted, which met the FIPS140-2 Level 3 of Federal Information Processing Standard [25].

## Results

### General information of people aged 50–75 years undergoing the FOBT for the first time

The results of the descriptive statistical analysis are provided in Table 1. Between 2010 and 2013, a total of 2,488,864 people underwent FOBT for preventive health care, and 191,671 (7.7%) individuals tested positive. Among them, 1,412,702 (56.8%) were women. Regarding the medical institution where the screening was performed, most individuals underwent screening in northern Taiwan, and the least number of people (7,668 people or 0.3%) underwent screening in offshore island cities or counties. The mean age of the individuals who were screened for the first time was 58.9 years. The participants predominantly belonged to the age groups of 51–55 and 56–60 years, accounting for 679,984 people (27.3%) and 660,560 people (26.5%), respectively. Regarding the screening location, most people (1,935,212, 77.8%) were screened as outpatients. As for the medical unit category where the screening was performed, the number of subjects who underwent outpatient testing was 2,436,071 (97.9%), which was higher than that of the inpatient testing. Finally, regarding family history of CRC, most did not report a history (2,269,198, 91.2%).

**Table 1:** General information of people aged 50–75 undergoing the FOBT.

Variables	Categories	N	%	Mean ± SD
**Total**		2,488,864	100	
**FOBT result**				
	Negative	2,297,193	92.3	
	Positive	191,671	7.7	
**Sex**				
	Male	1,076,162	43.2	
	Female	1,412,702	56.8	
**Medical institution region where screening was performed**				
	Offshore cities and counties	7,668	0.3	
	Northern region	1,135,051	45.6	
	Central region	560,391	22.5	
	Southern region	718,091	28.9	
	Eastern region	67,663	2.7	
**Screening age**				58.9±6.3
	50	192,265	7.7	
	51–55	679,984	27.3	
	56–60	660,560	26.5	
	61–65	527,245	21.2	
	66–70	331,548	13.3	
	71–75	97,262	3.9	
**Screening location**				
	Community or workplace screening station	490,129	19.7	
	Outpatient	1,935,212	77.8	
	Inpatient	27,315	1.1	
	Other	362,028	1.5	
**Category of the medical unit where screening was performed**				
	Outpatient	2,436,071	97.9	
	Inpatient	52,793	2.1	
**Family history of CRC**				
	No	2,269,198	91.2	
	Yes	150,898	6.1	
	Unknown	68,768	2.8	

### General information of the population with a positive FOBT result that underwent follow-up examination

The descriptive statistical analysis results are presented in Table 2. From 2010 to 2013, a total of 98,482 people were identified as having positive FOBT results and underwent follow-up examinations. Among them, 53,559 (54.4%) were men. Regarding location, most participants underwent the follow-up in northern Taiwan (42,141, 42.8%), and the least number of participants (273, 0.3%) underwent the follow-up in offshore island cities or counties. Regarding the age of the participants who were found to be positive, the number of people in the age groups of 51–55, 56–60, and 61–65 years were similar, and the number aged 71–75 years was the least at only 5,448 (5.5%). Most people preferred being screened as outpatients in terms of location (79,060, 80.3%). In terms of the choice of medical unit category for follow-up examination, the number of subjects who underwent outpatient follow-up examination was 87,273 (88.6%), which was higher than that of those who underwent inpatient examination. A total of 6,748 people (6.9%) reported a family history of CRC, and most of the participants (92,544, 94.0%) chose colonoscopy, and the least (983, 1.0%) chose double contrast barium enema plus flexible sigmoidoscopy as the follow-up examination method. Most (51,254, 52.0%) were diagnosed as having polyps, 9,955 (10.1%) were normal, and 4,280 (4.3%) were diagnosed as having CRC. Regarding follow-up time, most (54,801, 55.6%) completed the follow-up within 30 days and 9,190 (9.3%) people underwent the follow-up after 90 days. The mean follow-up time for people who tested positive for FOBT was 41.02 days.

**Table 2:** General information of the population positive for FOBT that underwent follow-up examination.

Variables	Categories	N	%	Mean ± SD
**Total**		98,482	100	
**Sex**				
	Male	53,559	54.4	
	Female	44,923	45.6	
**Medical institution region where the follow-up was performed**	Offshore cities and counties	273	0.3	
	Northern region	42,141	42.8	
	Central region	22,091	22.4	
	Southern region	31,471	32.0	
	Eastern region	2,506	2.5	
**Age tested positive**				59.99±6.46
	50	6,206	6.3	
	51–55	22,415	22.8	
	56–60	24,821	25.2	
	61–65	23,333	23.7	
	66–70	16,259	16.5	
	71–75	5,448	5.5	
**Screening location**				
	Community or workplace screening station	16,797	17.1	
	Outpatient	79,060	80.3	
	Inpatient	1,467	1.5	
	Other	1,158	1.2	
**Medical unit category where the follow-up examination was performed**				
	Outpatient	87,273	88.6	
	Inpatient	11,209	11.4	
**Family history of CRC**				
	No	89,480	90.9	
	Yes	6,748	6.9	
	Unknown	2,254	2.3	
**Examination method**				
	Colonoscopy	92,544	94.0	
	Double contrast barium enema plus flexible sigmoidoscopy	983	1.0	
	Other	4,955	5.0	
**Examination result**				
	Normal	9,955	10.1	
	Hemorrhoids	28,149	28.6	
	Ulcerative colitis	530	0.5	
	Polyp	51,254	52.0	
	CRC	4,280	4.3	
	Other	4,314	4.4	
**Follow-up time**				41.02±40.65
	Within 30 days	54,801	55.6	
	31–60 days	26,067	26.5	
	61–90 days	8,424	8.6	
	91 days or longer	9,190	9.3	

### Differences in mean follow-up time in people with positive FOBT results who have undergone follow-up examination

Regarding medical institution region where the follow-up was performed, the mean follow-up time for the offshore region was the longest (50.63 days). The mean follow-up time for the five regions exhibited significant differences (p < 0.001). As for age at being tested positive, the mean follow-up time exhibited significant differences among the age groups (p < 0.001). Regarding screening location, inpatients had longer mean follow-up time, and the various categories exhibited significant differences (p < 0.001). With regards to the medical unit category where the follow-up examination was performed, there was a significant difference in mean follow-up time between people with positive FOBT results who underwent outpatient follow-up and those who underwent inpatient follow-up (p < 0.001). Regarding family history of CRC, significant differences were identified in the mean follow-up time among the three categories (p < 0.001). As for follow-up examination methods, the follow-up time for people receiving double contrast barium enema plus flexible sigmoidoscopy was longer (46.94 days), and significant differences were observed in the mean follow-up time among the three methods. Finally, regarding diagnosis results, the mean follow-up time for CRC was 39.25 days, and the mean follow-up time for each category exhibited significant differences (p < 0.001; Table 3).

**Table 3:** Differences in mean follow-up time in people with positive FOBT results who have undergone follow-up examination.

Variables	Categories	Mean follow-up period	
		Mean ± SD	p-value
**Total**	98,482		
**Sex**			
	Male	40.96±40.814	
	Female	41.08±40.454	0.659
**Medical institution region where the follow-up was performed**			
	Offshore cities and counties	50.63±37.689	
	Northern region	39.84± 38.623	
	Central region	39.25±40.550	
	Southern region	43.70±42.898	
	Eastern region	41.60±44.094	<0.001
**Age tested positive**			
	50	40.38±39.619	
	51–55	40.94±41.067	
	56–60	41.53±41.847	
	61–65	41.35±41.343	
	66–70	41.90±41.455	
	71–75	35.69±26.168	<0.001
**Screening location**			
	Community or colon screen stations	43.29±41.809	
	Outpatient	40.15±39.975	
	Inpatient	57.93±50.862	
	Other	46.07±47.860	<0.001
**Medical unit category where the follow-up examination was performed**			
	Outpatient	41.20±40.515	
	Inpatient	39.63±41.661	<0.001
**Family history of CRC**			
	No	41.17±40.934	
	Yes	39.04±38.762	
	Unknown	40.70±34.192	<0.001
**Examination method**			
	Colonoscopy	41.06±40.597	
	Double contrast barium enema plus flexible sigmoidoscopy	46.94±39.887	
	Other	39.07±41.652	<0.001
**Examination result**			
	Normal	41.05±39.596	
	Hemorrhoids	40.31±40.576	
	Ulcerative colitis	44.27±43.120	
	Polyp	41.49±40.731	
	CRC	39.25±40.925	
	Other	41.24±41.864	<0.001

### Factors affecting the mean follow-up time

Table 4 presents the model of the mean follow-up time of the participants. After sex was controlled, the mean follow-up time of people who tested positive and whose medical institution where the follow-up was performed was in an offshore island city and county was 10.079 days longer than that in the eastern regions. Further, each age group had a longer mean follow-up time than the age group of 71–75 years. Regarding screening locations, people who tested positive as inpatients had a follow-up time longer by 12.143 days compared to the reference group. With regards to category of the medical unit where the follow-up examination was performed, the mean follow-up time for people with positive FOBT results who underwent inpatient follow-up was 1.257 days shorter than that in those who underwent outpatient follow-up. Compared with that of people not knowing their family history, the mean follow-up time of people with a family history of CRC was 2.562 days shorter. As for the follow-up examination method, people who tested positive and then underwent colonoscopy and those who underwent double contrast barium enema plus flexible sigmoidoscopy had longer mean follow-up time compared with people undergoing other examination methods; their mean follow-up time were 2.488 days and 8.902 days longer, respectively. Regarding diagnosis, people with hemorrhoids or with CRC had shorter mean follow-up time compared with those for people with other results; their times were shorter by 1.578 and 2.476 days, respectively.

**Table 4:** Factors affecting the mean follow-up time in those who tested positive for FOBT.

Variables	Categories	B	Standardized coefficients	p-value
**Constant**		38.936		<0.001
**Sex**	Male (reference group)			
	Female	0.279	0.003	0.290
**Medical institution region where the follow-up was performed**	Eastern region (reference group)			
	Offshore cities and counties	10.079	0.013	<0.001
	Northern region	−0.605	−0.007	0.471
	Central region	−1.374	−0.014	0.110
	Southern region	2.932	0.0.34	<0.001
**Age tested positive**	71–75 (reference group)			
	50	4.778	0.0.29	<0.001
	51–55	5.289	0.055	<0.001
	56–60	5.831	0.062	<0.001
	61–65	5.554	0.058	<0.001
	66–70	6.082	0.056	<0.001
**Screening location**	Other (reference group)			
	Community or workplace screening station	−2.675	−0.025	0.030
	Outpatient	−5.355	−0.052	<0.001
	Inpatient	12.143	0.036	<0.001
**Category of the medical unit where the follow-up examination was performed**	Outpatient (reference group)			
	Inpatient	-1.257	-0.010	<0.002
**Family history of CRC**	Unknown (reference group)			
	No	−0.579	−0.004	0.509
	Yes	−2.562	−0.016	0.010
**Examination method**	Other (reference group)			
	Colonoscopy	2.488	0.015	<0.001
	Double contrast barium enema plus flexible sigmoidoscopy	8.902	0.022	<0.001
**Examination result**	Other (reference group)			
	Normal	−0.477	−0.004	0.522
	Hemorrhoids	−1.578	−0.018	0.022
	Ulcerative colitis	2.452	0.004	0.191
	Polyp	−0.294	−0.004	0.664
	CRC	−2.476	−0.012	0.006

R^2^ = 0.008, Adjusted R^2^ = 0.007.

## Discussion

### General information

The database analysis revealed that the mean age of the people who underwent the FOBT for the first time for preventive health care in Taiwan was 58.9 years, which is higher than the 50 year age limit recommended for cancer screening. Regarding the screening location and category of the medical unit, people who underwent the FOBT tended to be outpatients. As FOBT is a preventive health insurance service in Taiwan, the eligibility of the subject will be noted in the health insurance card. The hospitals or primary care clinics providing FOBT are all under the contract of the National Health Insurance to participate in the colorectal cancer screening program [8]. Therefore, when an individual goes for outpatient consultation, the screening status will be indicated in the health care system, and the health care provider will inform the individual regarding the screening information. The health insurance system will also prompt if hospitalized patients meet the screening criteria, and medical staff will carry out screening after considering the patient’s condition and intention. The medical laboratory department in the hospital will process the fecal samples for analysis, whereas clinics will commission medical laboratories for testing. The follow-up of cases with positive FOBT results is the responsibility of case managers in the hospital and nurses in the clinic. In addition, the number of primary clinics has increased from 2010 to 2013, reaching 22,333 in 2018 [28–32], which affects people’s willingness to undergo screening. Therefore, the accessibility and convenience of qualified clinics has made them the location of choice for screening for many people. Moreover, health units in each administrative region cooperate with local medical institutions and go to communities or workplaces to offer cancer screening services to facilitate the testing of community dwellers and working people (Table 1). The number of people who tested positive for FOBT and underwent a follow-up examination was 98,482. The numbers of men and women were similar. The age of the participants was concentrated between 51 and 65 years, and most did not have a family history of CRC, which was similar to the results of relevant studies [33,34]. Most of the institutions that the participants went to were in metropolitan areas, such as the northern, central, and southern regions; this was possibly affected by the distribution of medical resources and education level [9,27]. The follow-up examination method most people underwent was colonoscopy. If patients test positive for FOBT, the patient should be followed up by the physicians belonging to the departments of colon and rectal surgery, endoscopy, gastrointestinal internal medicine, or general surgery to understand the cause of the FOBT for positive [8,15]. The standard, comprehensive recheck methods are colonoscopy or double contrast barium enema plus flexible sigmoidoscopy [8,15,35]. Most people were diagnosed as having polyps or hemorrhoids; 4.3% had CRC. Clearly, among this group, a follow-up examination can detect colorectal-related symptoms and enable early treatment. In this study, the mean follow-up time for people who tested positive was 41.02 days. Approximately 82% of people completed the follow-up within 60 days. Another study suggested that the mean follow-up duration should be within 90 days but discovered it to be 112 days [9]. Other studies have found that the mean waiting period between the positive FOBT result and colonoscopy was 105–202 days [36,37]. In this study, 88.6% of people with positive FOBT results underwent follow-up examination in outpatient clinics and 95% underwent colonoscopy or double contrast barium enema plus flexible sigmoidoscopy. This is due to a well-established colorectal cancer screening program that includes determination of a FOBT screening group, wide availability of screening sites, actively contact of positive individuals, and high accessibility to specialty care and follow-up [8,15,28–32]. Relevant studies have shown that approximately 25%–59% of people complete examinations, such as colonoscopy or double contrast barium enema plus flexible sigmoidoscopy, in the 1-year follow-up period [8,35,38], which suggests that Taiwan has a favorable result for the follow-up examinations after a positive CRC screening result (Table 2).

### Differences in mean follow-up time

A study revealed that among people who tested positive for FOBT, approximately half had a follow-up time affected by medical institutional factors [9]. In this study, the longer mean follow-up time of offshore cities and counties is likely a result of less medical resource allocation [27]. The mean number of days to follow-up examination for each age group was approximately 40. However, the mean number of days to follow-up examination for the group aged 71–75 years was 35 days as most people in this age group are retired and can easily arrange a time to undergo colonoscopy after conversing with medical personnel. In contrast, people of other age groups may be working and may be unable to easily arrange a time for undergoing the procedure. Regarding screening location, inpatients had a longer mean follow-up time compared to the reference group, possibly because they were in the hospital because of other illnesses and could not immediately undergo colonoscopy. In terms of the category of the medical units performing follow-up examination, those with positive FOBT results will be evaluated by an outpatient specialist and will be asked to undergo colonoscopy, with confirmed dietary control and purgative administration prior to the test [8,15], so that colonoscopy can be performed on the day of the appointment in the outpatient clinic. Therefore, the time from notification of positive FOBT results to the completion of follow-up examination is long [9,36,37]. For inpatients with positive FOBT results, the physician will consider their condition and adopt examination methods with lower risk [15]. In addition, when inpatients test positive for FOBT, the physician will evaluate and arrange for follow-up examination, where it can be reserved on the day of examination to reduce the waiting time. Therefore, the follow-up time for inpatients with positive FOBT results is shorter than that for the outpatients. Analysis in terms of family history of CRC revealed that positive cases with a family history of CRC had a shorter mean follow-up time compared with its two other counterparts possibly because they had knowledge about the disease and were more proactive in subsequent follow-ups. Regarding examination methods, Taiwan adopts double contrast barium enema plus flexible sigmoidoscopy as the assistive examination method for people who cannot undergo colonoscopy. In implementation, both items must be conducted to be considered a comprehensive colorectal test [8]. Therefore, the mean follow-up time for double contrast barium enema plus flexible sigmoidoscopy was longer. The mean follow-up time for those with positive screening for FOBT varied significantly among diagnoses, and related studies show that there is effective control of the follow-up time for those with positive screening results for FOBT [18–20]. In this study, location of the institution where the follow-up examination was performed, age, screening location, category of follow-up institution, family history, follow-up examination method, and diagnosis all exhibited significant differences in mean follow-up time (Table 3). Relevant studies have discovered that medical resources and health care system, patient factors, screening process and cancer diagnosis method, and health policies may affect the willingness of 40%–85% people who test positive for FOBT to undergo a follow-up examination [9,19,21,39,40].

### Factors affecting mean follow-up time

Factors affecting the mean follow-up time of the participants consisted of the region of the follow-up examination institution, age, screening location, category of the medical unit where follow-up examination was performed, family history, examination method, and diagnosis (Table 4). In offshore cities and counties where medical resources were scarce, follow-up examinations were delayed. Compared with the eastern region, people in the southern region waited 2.932 days longer. According to the Yearbook of Manpower Survey Statistics of 2013, more people in the southern region aged 50 years or over work than do their counterparts in the eastern, central, and northern regions [41]. Related studies showed that there was no significant correlation between the socioeconomic status of those who screened positive for FOBT and those who underwent follow-up examination, but there was a significant increase in compliance with follow-up testing among those with higher socioeconomic status [9].The results of another study showed that people with low socioeconomic status face a large number of obstacles in undergoing colonoscopy examinations, including pre-examination dietary management, purgative administration, withdrawal of anticoagulants, leave application, and job nature [8,15,42,43], which also indirectly affects the willingness of the working class to undergo follow-up examinations. With regards to age, the mean follow-up time in people with positive FOBT results aged 50–70 years was higher than that of the reference group. A related study showed that the ratio of people with positive FOBT results who undergo follow-up examination is higher in those aged 50–59 and 70–75 years [9]. In Taiwan, 2,523‰ and 219‰ of the working population are aged 50–64 and >65 years, respectively [41]. With regards to scheduling for undergoing colonoscopy, the group aged 50–70 years who may are still working need to consider pre-examination preparation time and the nature of their work compared with the group aged 71–75 years who may are not working [8,15,42,43], which may lead to a longer follow-up time. As for screening location, people who test positive in a community or workplace screening station or in an outpatient setting are less likely to have a severe illness; thus, arranging for colonoscopy for them is easy. In contrast, most inpatients have diseases or may have conditions with high severity. As a result, arranging colonoscopy requires the assessment of their disease and related factors, and hence, their mean follow-up time may be lengthier compared to the reference group. With regard to the category of the medical unit where follow-up examination was performed, after a physician has confirmed that an inpatient with positive FOBT results can undergo follow-up examination, an auxiliary examination with lower risk, such as double contrast barium enema or flexible sigmoidoscopy, may sometimes be selected to decrease examination inconvenience [15]. In addition, this can decrease the waiting time. Therefore, the waiting time for inpatients with positive FOBT results is lower than that of the outpatients. As for family history of CRC, people with a family history who tested positive were proactive in receiving follow-up examinations, and their follow-up time was short. Regarding examination method, currently, colonoscopy is the most comprehensive examination tool [8,15,35]. Therefore, after evaluation, most physicians will recommend undergoing colonoscopy or double contrast barium enema plus flexible sigmoidoscopy. As these cases require dietary control and bowel cleansing medication prior to screening [8,15], there is a long pre-procedure period. Moreover, double contrast barium enema plus flexible sigmoidoscopy can be performed on different dates and the date of the final examination is defined as the follow-up completion date. Therefore, the follow-up time is longer. Other examinations will usually employ flexible sigmoidoscopy or double contrast barium enema alone as alternative methods. Flexible sigmoidoscopy can be immediately performed after an enema; however, the purgative should be drunk in advance for double contrast barium enema [15]. Therefore, comprehensive colon examination methods have a longer follow-up time than that of the other examination methods. The diagnosis results indicating hemorrhoids or CRC had shorter mean follow-up time, revealing that relevant colorectal diseases and cancer can be targeted for early treatment through screening, follow-up, and diagnosis. Empirical data indicate that CRC is a type of cancer that can be detected and treated in its early stage through preliminary screening and the discovery of abnormalities. When it is discovered early, people with CRC who undergo proactive treatment have a 90% survival rate. In contrast, only 10% of people with late stages of CRC have a 5-year survival rate [9,44,45].

To summarize, the follow-up time of people who tested positive for FOBT was related to disease development. A relevant study reported that, compared with people who underwent colonoscopy 8–30 days after they tested positive, people who underwent it within 6 months of testing positive did not have an increased risk for diseases related to CRC. However, people whose follow-up period exceeded 6 months had an increased risk for stage Ⅱ CRC. For people whose follow-up time reached 12 months, their disease evolved into stage Ⅱ–Ⅳ CRC and they also developed other related diseases [18]. Factors affecting the follow-up time include age, sex, ethnicity, personal health cognition, health education, follow-up method, the degree of physician intervention, and health policy [9,21,22,46–48].

The CRC screening database was collected and established by the public sector; thus, the data samples are credible and representative. However, the analysis of the secondary database has limitations. (1) This study lacked detailed personal health information on the screened population. Cancer screening behavior and disease follow-up examination involve multiple factors, such as educational level, socioeconomic background, marital status, dietary habit, exercise frequency, and whether one undergoes regular health examinations. These data cannot be obtained from the database; therefore, the research on relevant topics was limited. (2) Information in the “other” columns was unclear. Some of the research variables had the option of “other,” without supplementary descriptions, resulting in the loss of relevant data. (3) Specific disease data was lacking. The database did not have relevant information for disease groups, such as whether CRC was present before the screening and whether other types of cancer or other chronic diseases were present that may have led to the overestimation of cases positive for CRC. In addition, the colorectal cancer screening database has been recently established, and some of the fields are still undergoing follow-up and compiling. For example, undisplayed gender, missing date, wrong code, and omission of screening or follow-up institution can lead to underestimation of screening cases.

## Conclusions

The results of this study indicate that factors affecting the follow-up time of people who tested positive in the FOBT results were the region and category of the follow-up medical unit, age, screening location, family history, examination method, and diagnosis results. We believe that screening is most essential for cancer prevention. However, to date, the establishment and implementation of relevant policies for cancer prevention generally require medical personnel to take the initiative. In the future, people’s cognition and knowledge and their capability in personal cancer prevention should be increased, and they should reinforce self-health management. Therefore, to ensure screening at the appropriate age and to construct a comprehensive follow-up network, popularizing health education and having sufficient diagnosis tools are crucial in CRC prevention policies. The results of this study may serve as a reference for future studies on reducing the follow-up time of people who test positive for FOBT.
